# Breeding Brown Pelicans Improve Foraging Performance as Energetic Needs Rise

**DOI:** 10.1038/s41598-020-58528-z

**Published:** 2020-02-03

**Authors:** Brock Geary, Paul L. Leberg, Kevin M. Purcell, Scott T. Walter, Jordan Karubian

**Affiliations:** 1 Department of Ecology and Evolutionary Biology, Tulane University, 6823 Saint Charles Avenue, 400 Lindy Boggs Center, New Orleans, LA 70118 USA; 2 Department of Biology, University of Louisiana at Lafayette, 410 East St. Mary Boulevard, 108 Billeaud Hall, Lafayette, LA 70503 USA; 3 National Marine Fisheries Service, Southeast Fisheries Science Center, Beaufort Laboratory, Beaufort, NC USA; 4 Data Science and Analytics Group, Harrisburg University of Science and Technology, 326 Market St, Harrisburg, PA USA; 5 Department of Biology, Texas State University, 601 University Drive, San Marcos, TX 78666 USA

**Keywords:** Behavioural ecology, Food webs, Food webs, Ecosystem ecology, Ecosystem ecology

## Abstract

Optimal foraging theory states that animals should maximize resource acquisition rates with respect to energy expenditure, which may involve alteration of strategies in response to changes in resource availability and energetic need. However, field-based studies of changes in foraging behavior at fine spatial and temporal scales are rare, particularly among species that feed on highly mobile prey across broad landscapes. To derive information on changes in foraging behavior of breeding brown pelicans (*Pelecanus occidentalis*) over time, we used GPS telemetry and distribution models of their dominant prey species to relate bird movements to changes in foraging habitat quality in the northern Gulf of Mexico. Over the course of each breeding season, pelican cohorts began by foraging in suboptimal habitats relative to the availability of high-quality patches, but exhibited a marked increase in foraging habitat quality over time that outpaced overall habitat improvement trends across the study site. These findings, which are consistent with adjustment of foraging patch use in response to increased energetic need, highlight the degree to which animal populations can optimize their foraging behaviors in the context of uncertain and dynamic resource availability, and provide an improved understanding of how landscape-level features can impact behavior.

## Introduction

The efficiency with which animals acquire resources has fundamental implications for their survival and reproductive success. In turn, the demographic and evolutionary trajectories of populations are, among other factors, shaped by individual foraging outcomes, as those who maximize their energy acquisition relative to expenditure are likely to raise more offspring over their lifespans^1^. Foraging efficiency is predicated on the successful location of resources, and often varies when the distribution of those resources is subject to rapid change^2–4^. Moreover, animals’ energetic needs vary over time, which may favor those that adjust their behaviors appropriately^5,6^. The degree to which animals forage optimally has received considerable theoretical and empirical attention^7–10^. However, accurately characterizing the relationship between resource distribution and foraging performance in the context of shifting energetic requirements has proven difficult in natural systems because of challenges associated with accurately characterizing each relevant process in appropriate detail. This is particularly true for animals foraging at larger (e.g., several km^2^) spatial scales, which cannot be easily approximated in experimental settings^11,12^.

Abiotic conditions are important determinants of habitat quality, as they affect the availability of resources and are susceptible to rapid and unpredictable change in certain ecosystems^13,14^. Prey resources can be highly mobile, producing resource landscapes that are dynamic over both space and time^15,16^. These patterns are common in marine systems, where many top predator species feed upon small fishes that are locally abundant but have patchy spatiotemporal distributions due to currents and schooling behaviors, which move several tons of prey biomass in discrete units^17^. Central place foragers that must return to a home location between foraging bouts face particular challenges due to a combination of competition around the central place and limited knowledge of regional conditions due to constraints on their movements^18^. In such situations, it is thought that these populations must rely on some degree of initial environmental sampling and continually refine their understanding of resource distribution and consistency in relation to the central place over time^19,20^. In doing so, it is possible for individuals to meet their energetic needs if they are able to exploit patches where prey are more consistently available, even in landscapes that may be otherwise unpredictable^21^.

When making foraging decisions, energetic demands must be considered in tandem with uncertainty surrounding resource distributions^6,22,23^. Indeed, the magnitude of change in foragers’ energetic requirements over different life stages may inform strategies in a profound way^24^. Individuals often utilize prior knowledge and spatial memory to inform foraging strategies^25^, and may also employ specific tactics in anticipation of energetically demanding periods (e.g. molt^26^, migration^27^, hibernation^28^, breeding^29,30^) to maintain efficiency in the future^31,32^. However, while theoretical and experimental investigations of changing foraging behaviors^33–36^ and field-based assessments of predator habitat selection^32,37–39^ are common, studies examining how energetic demands impact the foraging behaviors of wide-ranging natural populations at fine temporal and spatial scales are rare^40,41^. Because these populations are likely to encounter complex patterns of environmental heterogeneity, obtaining this information will improve our understanding of the degree to which optimal foraging theory can describe animal behavior in dynamic environments.

The growing capacity to directly link habitat selection of prey with precise measures of predator behavior has the potential to improve our understanding of population-level foraging outcomes via individual-level behavioral processes. High-resolution data on environmental conditions and animal behavior are increasingly attainable^12,42^, presenting opportunities to directly infer changing foraging behaviors of predators in natural systems. If prey are sufficiently abundant, predators may randomly traverse landscapes without requiring any behavioral adjustments to meet their energetic needs^43,44^. Alternatively, if prey are more limited, predators may be driven to locate and establish fidelity to specific high-quality foraging areas unless they are excluded or resources are depleted^45,46^. Intermediate scenarios also exist in which predators may rely on knowledge of higher-quality regions, but search more randomly at fine scales within them^36,47^. Consistent with this last scenario, nesting birds have previously been found to prospect the environment in advance of^32^ or during the chick provisioning period^48^ to meet energetic demands at each developmental stage. Regardless of scenario, assuming that animals are foraging optimally in their environment, it would be expected that individuals’ behaviors should maintain, if not improve, the quality of the foraging patches they occupy over time as energetic demands increase, relative to background levels of resource availability.

In this study, we characterize dynamic relationships between predators and their environment to investigate the degree to which brown pelicans (*Pelecanus occidentalis*) track their primary prey, the Gulf menhaden (*Brevoortia patronus*), during a period of changing prey availability and energetic needs. This resource is regionally abundant and makes up over 95% of the brown pelican’s diet in the region^49^ but is distributed patchily in time and space^50^, and our data collection period corresponds to a period of increasing energetic demand for pelicans that is associated with the provisioning of growing chicks^49^. As environmental conditions that improve habitat quality for a prey species are likely to create foraging habitat for an associated predator in open water, we use a menhaden species distribution model to derive landscape-level insights into pelican foraging behavior over time. Because pelicans return annually to breeding areas, sometimes from hundreds of kilometers away^49,51,52^, many individuals likely begin nesting with only a very general knowledge of local marine conditions. Like many seabirds, these individuals must determine how to identify regions of high foraging patch quality and forage efficiently within them^47,53^. Our study colony supports large numbers of breeding brown pelicans, which suggests that prey is sufficiently accessible for many nests to be successfully fledged. For these reasons, we hypothesized that increased energetic requirements associated with provisioning of young would drive breeding pelicans to occupy higher-quality foraging patches over time. Specifically, we predicted a relative increase in foraging habitat quality over time that exceeds changes in background landscape levels.

## Results

**Figure 1:**
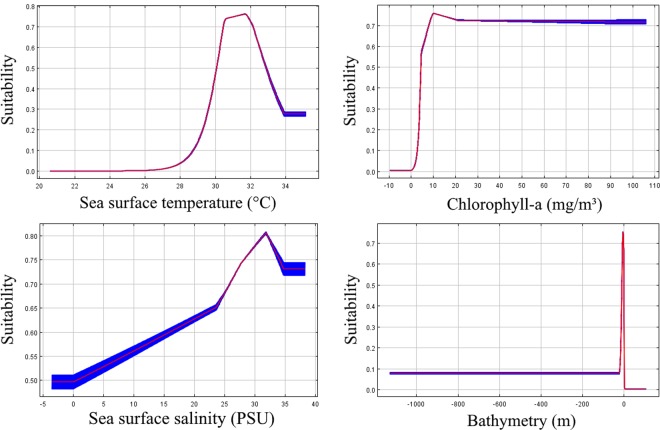
Maxent response curves for each environmental variable used to determine menhaden presence. Blue shading represents ±1 standard deviation from the mean.

The final averaged species distribution model linking environmental conditions to menhaden presence possessed an AUC value of 0.877 ± 0.004, indicating good predictive ability. Sea surface temperature and chlorophyll-a were the most important contributors to foraging habitat quality (Table 1). More specifically, we observed a sharp rise in suitability between 30–32 °C and a plateau of suitability at chlorophyll-a values ≥ 10 mg/m^3^ (Fig. 1). While the model specified slight optima for the other variables, contributions from both bathymetry and salinity were low (Table 1).

**Table 1:** Environmental variables (with sources) used in Maxent models, and variable importance for each layer.

Variable (units)	Data Product	Source	Percent Contribution	Permutation Importance
Sea surface temperature (°C)	NASA MODIS Ocean Aqua OceanColor	Movebank Env-DATA	47.1	54.5
Chlorophyll-a (mg/m^3^)	NASA MODIS Ocean Aqua OceanColor	Movebank Env-DATA	47.0	30.2
Sea surface salinity (PSU)	NOAA National Centers for Environmental Information	World Ocean Database and World Ocean Atlas Series	5.5	14.6
Bathymetry (m)	NOAA National Centers for Environmental Information	ETOPO1 Global Relief Model	0.3	0.8

**Figure 2:**
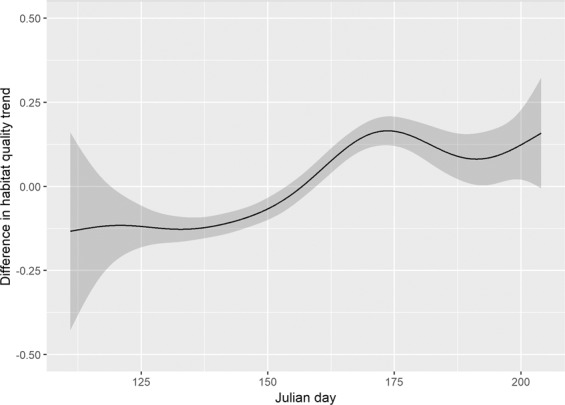
Pointwise difference estimates, with 95% confidence interval, between smooths of observed vs simulated foraging pelican efficiency based on generalized additive model output. Pelican foraging efficiency was lower than that of random simulated points at the beginning of the tracking period, but improved to the point that it was significantly higher for ~25% of the study.

**Figure 3:**
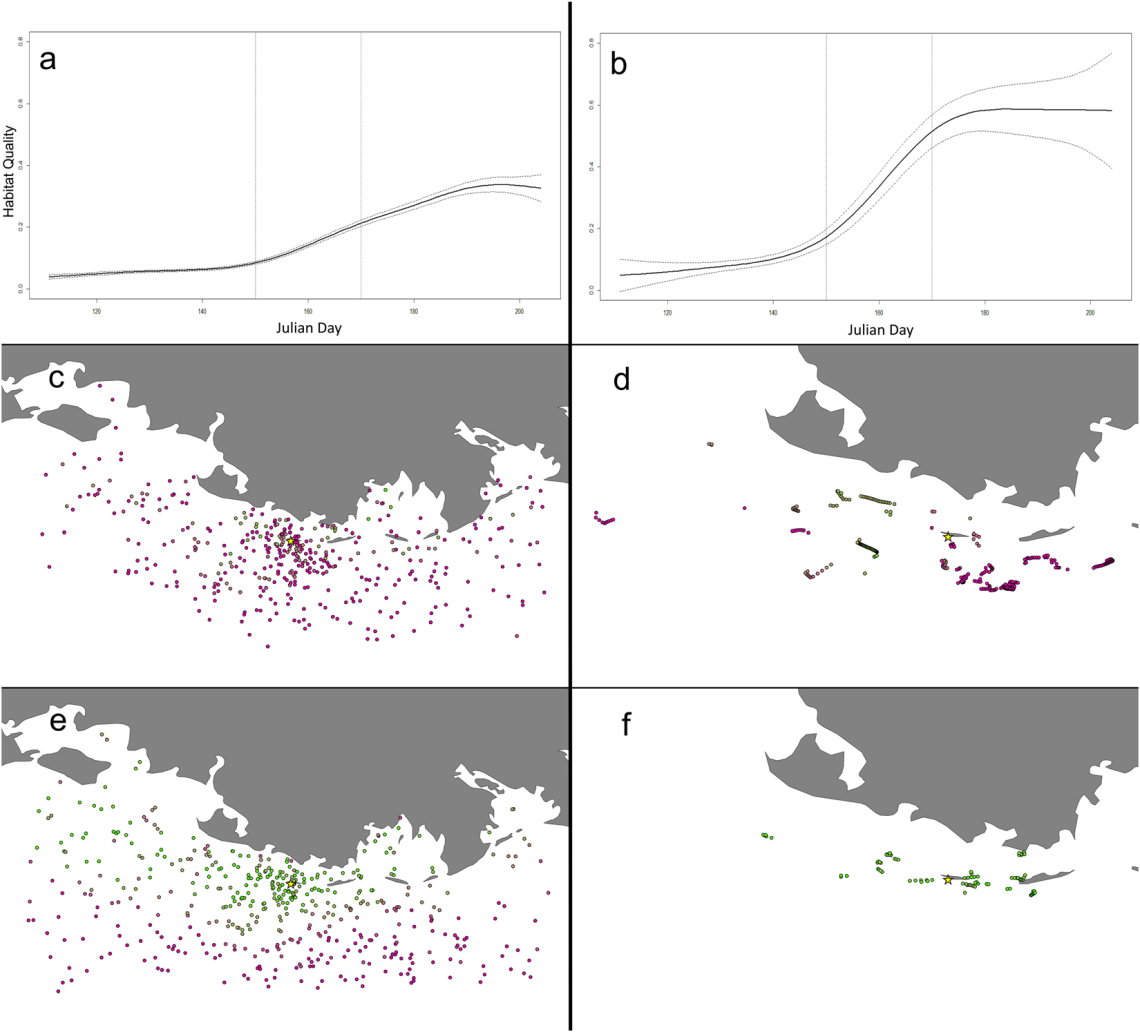
Generalized additive model curves demonstrating trends in foraging habitat quality, comparing regional changes over time (**a**) with brown pelican foraging locations (**b**). Representative maps of habitat quality (gradient of purple/low to green/high) change in the regional (**c**,**e**) and pelican (**d**,**f**) data sets show three-day windows surrounding Julian days 150 (**c**,**d**) and 170 (**e**,**f**) to capture a period of pronounced change marked in (**a**,**b**) by vertical lines. The star in each map denotes the position of the colony on Raccoon Island.

We found no significant relationships between foraging habitat quality and individual pelican characteristics (all p > 0.05). All predictors of foraging habitat quality in the additive model were highly significant (p < 0.001; Table 2), with 51.1% deviance explained by the model, and over 99% of which was explained by the model’s fixed effects. We observed significant overall annual variation in average habitat quality, and all years were significantly different from one another except for 2014 and 2017 (Wald test; all X^2^ > 16, df = 1, p < 0.001). Habitat quality within years generally increased over time in an S-shaped pattern: pelicans occupied significantly poorer foraging habitat than random simulated points early in the season, but improved more quickly than did simulated locations, to the point that they occupied significantly better habitat for approximately 25% of the tracking period (Fig. 2). This corresponded to a more restricted spatial distribution of foraging locations over time (Fig. 3, Supplementary Material), which in addition to the use of consistently high-quality areas, also included visitation to areas of more variable habitat quality, but only when quality was high (Supplementary Material).

**Table 2:** Generalized additive model output for comparisons of GPS and simulated pelican foraging locations over time.

Variable	Estimate	Standard Error	t value	p-value
Group (Pelicans)	0.883	0.054	16.378	<0.001
Year (2015)	0.282	0.030	9.360	<0.001
Year (2016)	0.098	0.031	3.127	0.002
Year (2017)	−0.046	0.030	−1.491	0.136
**Smooth Terms**		**EDF, RDF**	**F value**	**p-value**
Day (GPS pelican points)		7.070, 8.091	4790.245	<0.001
Day (Simulated pelican points)		4.553, 5.577	430.868	<0.001

Estimates for parametric terms represent increases in log-odds while holding all others constant. EDF = estimated degrees of freedom and RDF = residual degrees of freedom for additive model terms.

## Discussion

While many studies of predator foraging ecology take habitat preferences into account, few have investigated how well these preferences match prey distributions in natural contexts. Consequently, uncertainty persists about the degree to which individuals may be over- or under-performing relative to prey availability at the landscape level. This study refines our existing understanding of seabird foraging ecology by characterizing daily foraging habitat quality of individual wild animals at high spatial and temporal resolutions. By using the environmental associations of a major prey item to describe foraging performance, we found that breeding brown pelicans dynamically adjusted their foraging locations over time to improve occupied patches at a rate that exceeded background rates of change, presumably allowing them to forage in areas of increased prey availability. We also found that these adjustments do not result in the convergence of foragers on one optimal location, as birds continually visited multiple locations along the Louisiana coast, utilizing areas of consistent high quality near the Isle Dernières barrier island chain (which includes the breeding colony), as well as those of more variable quality to the east and west, but only at times when that quality was high. This indicates a scenario in which prey are somewhat limited, but suggests that pelicans are able to forage in a hierarchical fashion in order to exploit specific ephemeral patches in more regionally reliable areas.

Several non-mutually exclusive mechanisms might be responsible for the increasingly optimized foraging we observed. First, we consider improvement of individual-level landscape knowledge and subsequent specialization of foraging behaviors by individuals. For animals that seek resources in a spatially hierarchical fashion, specific cues associated with the presence of resources are assessed once individuals arrive in broader regions that they consider to be of sufficient quality^53^. Individuals may also encounter these cues at variable rates or times, which can produce strategies of similar efficiency that utilize different tactics, possibly explaining the lack of observed differences in foraging habitat quality among individual birds. In keeping with this scenario, we previously reported differences in foraging site fidelity associated with varying degrees of exploration among individual birds, which may be associated with diverse foraging tactics^54^. Utilization of different bay regions could expose individuals to potential site-specific cues of equivalent value that they might use to the exclusion of others, including landscape features, the presence of other predators of menhaden such as marine mammals (which may more directly assess the environmental conditions used in our analyses), or commercial fishing vessels. We also observed differences in average regional habitat quality among most years, suggesting additional variation due to broader ecological processes that may shape menhaden distributions and the strategies needed to locate them. Further exploration of differential success in actual prey capture, in relation to variation in both cues and environmental conditions, will provide further understanding of the specific behaviors employed by successful foragers in this species.

Availability of conspecific social information is another potential mechanism that could explain our findings that behavioral ecologists have long invoked as a benefit of colonial living^55^. Information may be gained at the colony, where individuals are able to assess the departure directions, nesting status, and nestling provisioning of neighbors, or on foraging grounds, where both followers and leaders may benefit from local enhancement^56^. In the absence of previously acquired information, the proliferation of information through members of a colony should lead to improved foraging performance over time. This tactic may help to meet the energetic needs experienced by nesting pelicans, many of which migrate into the region to breed^51^ and lack recent information on prey distributions, and the rate at which correct information is obtained could be an important determinant of reproductive success, as nests that fledge multiple chicks are relatively infrequent in this population^57^.

One unexpected finding of this study is that brown pelicans began the tracking period by foraging in areas of significantly lower quality than would be expected by random chance, as represented by our contrasting simulated foraging locations. The results of our species distribution modeling agree with known information about menhaden habitat selection with regard to thermal tolerance and preference for higher plankton densities^58,59^, so it is sensible to infer that the foraging landscape is of lower overall quality for pelicans at the beginning of our tracking period as menhaden begin moving from estuaries to the near shore after spawning^50^. In line with predictions, poor habitats may be used when individuals do not expect increased returns from relocating, resulting in some period where occupied patches do not improve. Pelicans also spend more time near the nest when defending small chicks^49^, which may limit their ability to assess neighbors or search for novel foraging areas. Alternatively, if energetic needs are lower early in the nesting period, individuals may be able to take advantage of this period of less demand to prospect the region and determine which areas are likely to improve with time. We have not observed changes in foraging trip distance and duration within a breeding season^54^, so nest attendance does not appear to seriously constrain adult movements, but the ability to spend more time elsewhere (e.g. loafing on beaches with other birds) as chicks develop may present opportunities to gather additional public information that impacts foraging decisions. As the sources of this information are common throughout the landscape, with Louisiana’s coast containing tens of thousands of breeding pelicans in addition to fishing vessels and other species, it is not surprising that performance over time would substantially improve for successful breeders.

This study highlights the importance of linking foraging behaviors directly with underlying estimates of resource distributions, rather than assuming optimality based on habitat selection, in order to more precisely characterize foraging performance in natural systems. In addition to providing broader insights into the processes influencing foraging ecology in wild populations, this methodology also has potential to identify more causal relationships between climatic or structural changes and population trends over time if habitat quality is impacted for species of concern. Continued advances in remotely-sensed environmental data collection and tracking technology, as well as application of these methods across other landscapes and taxa, will continue to increase the detail with which these ecological processes may be questioned and examined, further refining our understanding of animal movements and optimal foraging theory in nature.

## Methods

### Menhaden species distribution modeling

We obtained data on Gulf menhaden distributions from 2012 Captain’s Daily Fishing Reports submitted by commercial fishing vessels to the National Marine Fisheries Service’s Beaufort Laboratory. In this database, which is believed to comprise nearly 100% of commercial harvest^60^, GPS locations and time stamps of landings (i.e. precise presence records of large menhaden schools) are recorded. We annotated all points that coincided in time (by Julian days) with our yearly pelican tracking periods with interpolated (inverse-distance weighted^61^) environmental data (sea surface temperature and chlorophyll-a; Table 1) using the Env-DATA service provided by the Movebank database, which performs interpolations over space as well as time using multiple layers of each variable^42^. We also annotated sea surface salinity and bathymetry data from interpolated rasters (Table 1) using ArcMap version 10.1 (ESRI, Redlands, California, USA). We chose these data based on their previous recognition as important variables to menhaden habitat suitability^58,59^. Using these annotated points, we used species distribution models generated by maximum entropy methods in Maxent version 3.4.1 (www.cs.princeton.edu/~schapire/Maxent/)^62,63^; to quantify the probability of menhaden presence for real and simulated pelican foraging points. Using the previously described environmental data, we generated a Maxent model using 10,000 annotated background points, 10-fold cross-validation and a regularization multiplier of 2 to avoid overfitting^64^, and 1,000,000 maximum iterations to ensure model convergence.

We evaluated model performance using the area under the curve (AUC) value, which represents the probability of a random presence value being assigned a higher suitability value than a randomly selected background point. AUC values can range from 0–1, where a value of 0.5 indicates that model predictions are no better than random, a value of 1.0 indicates perfect model fit, and values > 0.7 are considered to be indicative of good predictive ability^65,66^. We also assessed the importance of each environmental variable using two values: their percent contribution (based on amount of gain increase attributed to the variable during model fitting) and permutation importance (based on decrease in AUC when background data values for that variable are randomly permuted)^62^. We obtained an average AUC value across the 10 model runs (following the cross-validation procedure) and used the averaged model output to derive final suitability values, which roughly correspond to the probability of menhaden presence (complementary log-log or “cloglog” output, 0–1).

### Pelican data collection

We captured all brown pelicans during their nesting seasons on Raccoon Island, one of four barrier islands comprising the Isles Dernières chain located in Terrebonne Bay, Louisiana in the northern Gulf of Mexico (29.0519°N, −90.9336°W). Raccoon Island is the largest seabird colony in the region, with 3000–5000 brown pelican nests initiated each year^67^. GPS tracking work began as nest incubation was largely completed throughout the colony, typically in late April or early May of each year, for four consecutive years from 2014–2017. We tracked adults with nests constructed in black mangrove (*Avicennia germinans*) 1–1.5 m in height to control for potential differences in behaviors related to nest site selection^57^. We captured individuals either by hand or using leg snares and attached e-Obs© tracking units (e-Obs Digital Telemetry, Gruenwald, Germany) using a backpack-style harness made of Teflon ribbon (Bally Ribbon Mills, Bally, Pennsylvania, USA) and copper clasps. The full tracking apparatus weighed approximately 110 g, less than 5% of any bird’s body mass (range: 2600–4330 g). We took blood samples to sex individuals using lab-based methods^68^ and used morphological measurements to calculate indices of body condition for each sex (standardized residuals from linear regressions of log-transformed body mass on 3 * log(tarsus length)^69^. Tracking units recorded and stored GPS locations every 15 minutes during daylight hours. We revisited the island every 7–10 days following deployment, remotely downloaded tracking data to a handheld base station, and checked nests of tracked individuals to ensure that collected data represented behavior of birds that were continuing to provision young and therefore experiencing increased energetic demand. We continued tracking until chicks for each nest could no longer be located, at which point we assumed that they had either died or fledged. Across all years, raw data collection yielded 55,316 GPS points from 30 birds (mean = 1843.87 ± 884.20 (SD) pts/bird). We received usable round-trip data from 13 birds in 2014, 4 birds in 2015, 7 birds in 2016, and 6 birds in 2017 (n = 30 total birds), with an average of 41.77 (±17.80) tracking days per bird. All field protocols are in accordance with the relevant guidelines and regulations. We sampled all individuals under permit #06669 issued by the United States Geological Survey’s Bird Banding Laboratory, and handling protocols were reviewed and approved by the Tulane University Institutional Animal Care and Use Committee (#0395R2). All work was performed in accordance with relevant institutional guidelines and regulations. Pelican location data presented in this study were also previously published as part of a separate study: 10.1093/beheco/ary17354.

### Pelican data processing and analysis

We used Microsoft Excel and custom scripts in R version 3.5.1^70^ to remove duplicate locations and prepare data for analysis. We isolated ‘complete’ foraging trips that began and ended on the colony within the same day using the ‘adehabitatLT’ package in R^71^, removed locations on the nest or beach of the colony, and re-discretized each track to recover the entire trajectory of the foraging trip and to space locations to regular 200 m intervals.

**Figure 4:**
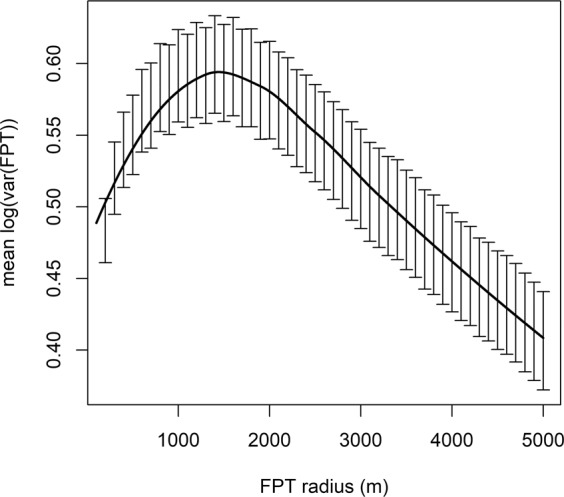
Trendline and 95% confidence intervals for each tested radius in first-passage time analysis. The peak mean log(FPT) value occurs at a radius of 1400 m.

To identify locations where tracked pelicans were most likely foraging, we utilized first-passage time (FPT) analysis to determine the scale at which pelicans were engaged in area-restricted search behavior^72^. This analysis is based on the assumption that animals transition to slower, less linear movements when foraging, relative to movements when transiting between sites^16^. We calculated FPT, or the time taken by an individual to traverse a circle of a given radius, along each foraging trajectory for a series of radius values (100–5000 m, at a 100 m interval), centered on each point to determine an optimum scale, defined as the radius at which the variance of log(FPT) is maximized. As the optimal FPT may vary slightly across individuals, we determined the overall optimum by averaging the maximum variance value of each radius for each individual, and used the largest of these values as a common spatial scale going forward^73^. This analysis revealed 1400 m as the optimum radius at which to detect area-restricted search behavior (Fig. 4). To identify the points within each foraging trip that represented area-restricted search behavior, we isolated the portions of each trip trajectory in the 90^th^ percentile of FPT values^74^, and extracted the corresponding points with their identifying date and time. We identified foraging points in 453 foraging trips from the 30 birds, yielding 4,915 locations for subsequent foraging habitat quality analysis (mean: 163.83 ± 112.27 locations/bird). To compare these locations to the overall landscape, we also generated 10,000 simulated foraging locations by randomly sampling (with replacement) time stamps, as well as angles and distances from the colony, from our foraging point data set. Using the final averaged output from the menhaden distribution model and interpolated environmental raster data from 2014–2017, we projected suitability values onto both the pelican and simulated data points, averaging values within pelican foraging trips to address likely autocorrelation. As areas that are most likely to contain menhaden can be interpreted as the highest-quality pelican foraging areas in the absence of confounding ecological factors, we hereafter refer to menhaden suitability values in our results as “foraging habitat quality” values, which serve as the response variable in subsequent analyses.

We performed all remaining statistical analyses in R. To examine among-individual variation in foraging habitat quality, we used a linear mixed-effect model in the package ‘nlme’^75^ that included sex, condition, and year as predictor variables, with individual bird identity as a random effect. We then used generalized additive modeling in the package ‘mgcv’^76^ to determine relationships between pelican foraging behavior and habitat quality over time. This model uses smooth functions of at least one covariate to model non-linear relationships with response variables of interest. For this analysis, we used foraging habitat quality for each trip as the response in a beta regression model (due to response values ranging 0–1), with year and real/simulated grouping as parametric model terms, Julian day as a smooth term using cubic regression splines, and bird identity as a random effect. We also specified an interaction between Julian day and grouping to fit separate curves to the presence and simulated data and examine differential change in habitat quality over time. We used a post-hoc Wald test to compare pairwise intercepts between years. As a final post-hoc test, in addition to assessing the significance of each resulting smooth term, we used a prediction matrix to compare fitted values of the two smooth functions across all Julian days in the model using the methods of Rose *et al*.^77^. To do so, we generated an approximate pointwise 95% confidence interval to identify and plot time points at which the two fitted smooths differed significantly from one another. As a post-hoc exploration of spatial and temporal trends of habitat quality, we plotted each data point from both real and simulated data sets using the package ‘plotly’^78^ to examine changes in foraging habitat quality simultaneously over space and time.

## Data accessibility

We will archive all raw telemetry data sets in the Movebank database, and make all data sets and associated statistical code necessary to replicate results available in FigShare at the time of this manuscript’s acceptance.

## Supplementary information

Supplementary Information.
