# Supplementary material for: Breeding Brown Pelicans Improve Foraging Performance as Energetic Needs Rise
Source: Sci Rep. 2020 Feb 3;10:1686. doi: 10.1038/s41598-020-58528-z (PMC6997155; doi:10.1038/s41598-020-58528-z)
Supplement: Supplementary file 1 — Supplementary Information. [file 41598_2020_58528_MOESM1_ESM.zip › SupplementReadme.pdf]

Breeding brown pelicans improve foraging performance as energetic needs rise

Brock Geary, Paul L. Leberg, Kevin M. Purcell, Scott T. Walter and Jordan Karubian

Supplementary Material

This document contains two attachments: interactive, three-dimensional plots of foraging habitat quality of brown pelicans tracked in this study, as well as habitat quality throughout the region (as represented by a simulated data set, see Methods section in the manuscript). Simulated background points demonstrate changes in landscape-level habitat quality (purple/low to green/high), while pelican points demonstrate improved performance as foraging distributions narrow to almost exclusively high-quality areas over the course of the season.
